# Partial volume correction of brain PET studies using iterative deconvolution in combination with HYPR denoising

**DOI:** 10.1186/s13550-017-0284-1

**Published:** 2017-04-21

**Authors:** Sandeep S. V. Golla, Mark Lubberink, Bart N. M. van Berckel, Adriaan A. Lammertsma, Ronald Boellaard

**Affiliations:** 1 Department of Radiology and Nuclear Medicine, VU University Medical Center, PO Box 7057, 1007MB Amsterdam, The Netherlands; 2 Department of Nuclear Medicine and Molecular Imaging, University Medical Center Groningen, University of Groningen, Groningen, The Netherlands; 3 Nuclear Medicine and PET, Department of Surgical Sciences, Uppsala University, Uppsala, Sweden

## Abstract

**Background:**

Accurate quantification of PET studies depends on the spatial resolution of the PET data. The commonly limited PET resolution results in partial volume effects (PVE). Iterative deconvolution methods (IDM) have been proposed as a means to correct for PVE. IDM improves spatial resolution of PET studies without the need for structural information (e.g. MR scans). On the other hand, deconvolution also increases noise, which results in lower signal-to-noise ratios (SNR). The aim of this study was to implement IDM in combination with HighlY constrained back-PRojection (HYPR) denoising to mitigate poor SNR properties of conventional IDM.

**Methods:**

An anthropomorphic Hoffman brain phantom was filled with an [^18^F]FDG solution of ~25 kBq mL^−1^ and scanned for 30 min on a Philips Ingenuity TF PET/CT scanner (Philips, Cleveland, USA) using a dynamic brain protocol with various frame durations ranging from 10 to 300 s. Van Cittert IDM was used for PVC of the scans. In addition, HYPR was used to improve SNR of the dynamic PET images, applying it both before and/or after IDM. The Hoffman phantom dataset was used to optimise IDM parameters (number of iterations, type of algorithm, with/without HYPR) and the order of HYPR implementation based on the best average agreement of measured and actual activity concentrations in the regions. Next, dynamic [^11^C]flumazenil (five healthy subjects) and [^11^C]PIB (four healthy subjects and four patients with Alzheimer’s disease) scans were used to assess the impact of IDM with and without HYPR on plasma input-derived distribution volumes (*V*_T_) across various regions of the brain.

**Results:**

In the case of [^11^C]flumazenil scans, Hypr-IDM-Hypr showed an increase of 5 to 20% in the regional *V*_T_ whereas a 0 to 10% increase or decrease was seen in the case of [^11^C]PIB depending on the volume of interest or type of subject (healthy or patient). References for these comparisons were the *V*_T_s from the PVE-uncorrected scans.

**Conclusions:**

IDM improved quantitative accuracy of measured activity concentrations. Moreover, the use of IDM in combination with HYPR (Hypr-IDM-Hypr) was able to correct for PVE without increasing noise.

**Electronic supplementary material:**

The online version of this article (doi:10.1186/s13550-017-0284-1) contains supplementary material, which is available to authorized users.

## Background

Positron emission tomography (PET) is a non-invasive molecular imaging tool for quantitative analysis of in vivo physiology. An important issue affecting the accuracy of PET quantification is its limited spatial resolution. The spatial resolution, characterised by the point spread function (PSF) of a PET scanner, is affected by several intrinsic (isotope dependent positron range [1], photon non-colinearity) and external (size of PET detectors, detector block design) factors. The limited resolution of PET results in the so-called partial volume effect (PVE), i.e. a decrease or increase in apparent activity concentration for regions that are in close (i.e. distance in the order of about two to three times the spatial resolution of the images) proximity to another region with lower or higher activity concentration, respectively [2]. The resulting spill-out and spill-in of activity between volumes of interest (VOI) give rise to inaccurate estimates of regional activity concentrations. A concise description of all factors involved in PVE can be found elsewhere [3, 4].

In theory, true PET activity concentrations can be restored by correcting for PVE. Most common approaches use anatomical information and tissue homogeneity constraints [4–7]. These approaches, however, require additional structural information, e.g. from magnetic resonance (MR) scans. In some cases, MR data are not available or not of sufficient or comparable (across subjects and/or studies) quality. This may occur when different MR systems are being used within a study. Moreover, besides this possible variability in the MR data quality, also the quality of the coregistration between MR and PET images and the performance of tissue segmentation methods affect the performance of PVC, as was recently demonstrated by Greve et al. [8]. An alternative approach for PVC is to either use deconvolution in the image domain [9, 10] or incorporate the PSF in an iterative image reconstruction algorithm [11, 12]. To date, not all PET/CT systems allow for incorporation of the PSF during on-line reconstructions and, therefore, a post reconstruction implementation might be of interest.

For post reconstruction PVE corrections, iterative deconvolution methods (IDM) have been used, such as the Van Cittert [9] and Lucy-Richardson [13] deconvolution methods. Unfortunately, these methods also result in noise amplification, resulting in poor signal-to-noise ratios (SNR). Noise control may be achieved by performing spatial filtering during or after reconstruction [14], but this reduces noise at the cost of lower resolution.

Another possible and more desirable way of reducing noise would be to use temporal regularisation of the IDM-corrected image. Sophisticated temporal regularisation techniques would ensure restoration of the PET images, while retaining a good SNR. Recently, Reilhac et al. [15] published a method to achieve PVC using IDM along with spatiotemporal regularisation. They were able to correct for PVE, while controlling statistical noise. The present study describes an alternative denoising technique called HighlY constrained back-PRojection (HYPR) [16–18]. HYPR allows for improvements in image SNR without notably affecting spatial resolution. The aim of this study was to develop and evaluate a partial volume correction (PVC) method based on iterative deconvolution in combination with HYPR denoising to mitigate poor SNR properties of conventional IDM.

## Methods

### Iterative deconvolution methods (IDM)

Van Cittert (VC) and Lucy-Richardson IDMs were evaluated for image PVC. Below is a short description of the VC IDM method.

In the VC algorithm, a copy of the original PET image is smoothed using a Gaussian-shaped kernel that represents the PSF of the scanner. Next, a difference image between this smoothed and the original PET image is generated. Next, the sum of this difference image and the original unsmoothed PET image results in a sharper image. This process is repeated using the sharpened image (as new original image). This process is repeated, and after each iteration, a PET image with a higher spatial resolution is obtained. The process may be stopped when further iterations would not further improve spatial resolution. However, iterative deconvolution also amplifies noise during each repetitive step, and often, the process is stopped prematurely to avoid poor signal-to-noise ratio. The following equations illustrate the IDM implementation:

1 $$ {\mathrm{EI}}_i={I}_i-\mathrm{S}\ {\mathrm{I}}_i $$

2 $$ {I}_{\mathrm{i}+1}={I}_{\mathrm{i}}+{\mathrm{EI}}_{\mathrm{i}} $$

where *I*_*i* + 1_ is the sharpened image, *I*_*i*_ is the original image, EI_*i*_ is the error image, and SI_*i*_ is the smoothened original image for the iteration *i*. Multiple iterations, *i*, are performed, and at each iteration, a new ‘sharper’ but ‘noiser’ estimate of the PET data is obtained.

In order to avoid noise amplification, two types of priors (median and Gibbs type) [14] were included in the IDM, each with a neighbourhood of 1, which could be assigned different contributing weights. The algorithm for using the prior is illustrated in Additional file 1 [19] by means of a pseudo-code. These priors were implemented to regularise IDM-induced noise amplification. In addition, we studied the impact of both prior types and several weight values on PVC performance. Finally, as IDM is an iterative technique, different numbers of iterations were tested. A 6.75-mm FWHM Gaussian PSF was used within IDM in this study. The FWHM was estimated based on the post reconstruction smoothing and was set such to meet EARL specifications in order to match the image resolution obtained from both systems used [20]. It should be noted that the PSF depends on the position in the scanner [13, 21]. Henceforth, an estimate of PSF was used in this study which might lead to some bias depending on the positioning of the object. However, in case of brain studies, the object is positioned near the centre of the scanner field of view and the effects of a non-stationary PSF were assumed to be small.

### HighlY constrained back-PRrojection (HYPR)

A HYPR image is defined as the product of a composite image and a weighting image, where the weighting image is computed as the ratio of the low-pass filtered original dynamic image and the low-pass filtered composite image. The composite image is the weighted average of a few or all frames of the dynamic 4D PET data set. Instead of summing (dynamic) images, the composite image can also be obtained by directly reconstructing it from a summed sinogram. The use of the composite image (low noise) results in improved SNR in the HYPR image. More details regarding HYPR can be found elsewhere [16, 17]. A Gaussian low-pass filter of 6.75-mm FWHM was used for HYPR. Equations 1–3 provide an insight into HYPR implementation.

3 $$ C={\displaystyle \sum {I}_i\times \varDelta {t}_i} $$

4 $$ {W}_i={\mathrm{SI}}_i/\mathrm{S}\mathrm{C} $$

5 $$ {I}_i\hbox{'}= C\times {W}_i $$

where *I*′ represents the HYPR image; *C* is the single-frame composite image which is the duration weighted average summation of *i* frames with ∆*t*_*i*_ duration; *I* represents the complete dynamic data; and *W* is the weighting images computed as the ratio of the low-pass filtered original dynamic image SI_*i*_ and the low-pass filtered composite image SC.

### Parameter estimation

IDM parameters such as the number of iterations [2, 4, 6, 8, 10], IDM with/without prior weight (0, 0.075, 0.15, 0.3 and 0.6), and IDM with/without HYPR were evaluated and optimised. Optimization was based on phantom studies, as activity concentrations are known and recovery coefficients can be calculated quantitatively.

### Order of implementation

IDM increases noise particularly in case of scan data with low statistics. Therefore, some of the previous studies have incorporated spatial noise regularisation post-IDM implementation [10]. In this study, HYPR was also tested instead of spatial priors. Furthermore, we combined IDM with HYPR denoising (instead of priors) in three ways: (1) IDM followed with HYPR (IDM-Hypr); (2) HYPR followed with IDM (Hypr-IDM); and (3) HYPR followed with IDM and followed again with HYPR (Hypr-IDM-Hypr).

### Phantom studies

An anthropomorphic brain Hoffman phantom with cylindrical dimensions of 17.5 (height) by 20.8 (diameter) cm and a fillable volume of 1.2 L was used. The phantom was filled with an [^18^F]FDG solution of ~25 kBq mL^−1^, and list mode data were acquired for 30 min using a Philips Ingenuity TF PET/CT scanner (Philips, Cleveland, USA) [22]. Data were reconstructed into 18 frames (6 × 5, 3 × 10, 4 × 60, and 5 × 300 s) using a blob-based, ordered subset iterative reconstruction algorithm (BLOB-OS-TF). This BLOB-OS-TF algorithm, as provided by the vendor, was implemented with default settings (matrix size 128 × 128 × 90; voxel size, 2 × 2 × 2 mm^3^; and a final image resolution of 6.75-mm FWHM).

A Hoffman phantom template (volume of interest indicating grey and white matter volume) was co-registered to the reconstructed images, and grey matter time activity curves (TACs) were extracted. Resulting TACs were compared with the true activity concentration both before and after implementation of IDM. Optimised parameters for IDM (such as number of iterations, type of algorithm, with/without prior weight, with/without HYPR) were selected based on the best average agreement between the resulting and actual concentrations in the grey matter.

### Clinical studies

The performance of IDM and HYPR was evaluated for clinical applications using two different tracers. [^11^C]Flumazenil ([^11^C]FMZ) is a central benzodiazepine receptor tracer with high cortical uptake, high statistics and high grey to white matter contrast. [^11^C]PIB (Pittsburgh compound B) is an amyloid tracer with low to intermediate grey to white matter contrast in AD subjects and with an inverse grey to white matter contrast in healthy subjects. Dynamic [^11^C]FMZ and [^11^C]PIB scans from previously reported studies [21, 23] were used in this study. [^11^C]PIB data were derived from four patients with Alzheimer’s disease (AD) and from four healthy subjects. The 90-min dynamic [^11^C]PIB scans consisted of frames with variable length (1 × 15, 3 × 5, 3 × 10, 2 × 30, 3 × 60, 2 × 150, 2 × 300 and 7 × 600 s). In addition, 60-min dynamic [^11^C]FMZ scans with variable frame length (4 × 15, 4 × 60, 2 × 150, 2 × 300, and 4 × 600 s) from five healthy subjects were included. Scans were acquired on an ECAT exact HR+ PET scanner (CTI/Siemens, Knoxville, USA). For each scan, first a 10-min transmission scan in 2D acquisition mode was obtained. Subsequently, a dynamic emission scan was performed following a bolus injection of ~370 MBq of the specific tracer. All [^11^C]PIB studies were reconstructed using ordered-subset expectation maximisation (OSEM) with four iterations and 16 subsets, and [^11^C]FMZ scans were reconstructed using FORE+2D filtered back projection. All dynamic scan data were corrected for detector normalisation, photon attenuation, decay, dead time, scatter and random coincidences. After reconstruction, all images were filtered using a 5-mm FWHM Gaussian filter and had a matrix size of 128 × 128 × 63, a voxel size of 2.42 × 2.42 × 2.42 mm^3^, and a final image resolution of 6.75-mm FWHM. All scans were approved by the medical ethic committee of VU University medical centre. Additionally, a written consent was given by each subject prior to inclusion in the study.

Both protocols included continuous arterial blood sampling using a dedicated on-line sampling system [24]. In addition, whole blood and plasma concentrations and labelled metabolite fractions were measured using manual samples collected at different preset time points. Finally, based on both on-line and manual blood data, metabolite-corrected plasma input functions were obtained. Volumes of interest (VOIs) were delineated on co-registered T1-weighted MR scans and transferred automatically onto the PET scans using PVElab [25] with Hammers template [26]. In the present study, regional distribution volume (*V*_T_) was obtained from both non-linear regression (NLR) analysis using a reversible two tissue compartmental model and Logan plot analysis [27]. Logan plot is a linearization method to obtain parametric images for reversible tracers and is one of the most frequently used linear methods. This technique involves linear transformation of the equations such that the slope of the line represents the *V*_T_. Logan images were used for visual interpretation and quantitative evaluation of the effect of IDM. Finally, regional *V*_T_ values obtained before and after implementation of IDM were compared with each other to assess the impact of IDM compared to data without any partial volume correction.

In addition, the impact on the noise of IDM was evaluated for the different techniques described. This assessment was performed using three representative clinical scans, i.e. one FMZ scan and both AD and HC PIB scans. A volume of interest was drawn within white matter (centrum semiovale), where the measured activity is nearly uniform, using the standard deviation of the voxel values within the volume of interest as metric for characterising image noise. To this end, the coefficient of variation (%) of the VOI voxel values was derived for each of the three scans and for each implementation (i.e. with spatial normalisation and Hypr_IDM_Hypr) and studied as function of frame midpoint time.

## Results

Although both VC and Lucy-Richardson IDMs were evaluated for image PVC, as both implementations are algorithmically similar and provided similar results, only results from the VC IDM implementation will be presented.

### Parameter optimisation

**Fig. 1:**
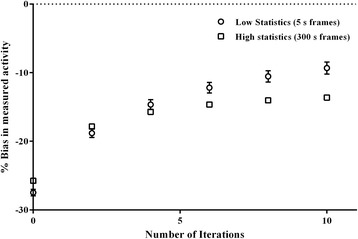
Effect of number of IDM iterations on bias (mean ± SD of all 5 s frames and 300 s frames) in measured activity concentrations in grey matter regions of the Hoffman phantom

Figure 1 illustrates the bias in measured activity concentrations in grey matter regions of the Hoffman phantom for IDM with varying number of iterations and for both high- and low-statistics data (frame durations). It is apparent that with increasing number of iterations, the relative differences tended to stabilise, and we considered eight iterations to be optimal. Another important aspect of IDM is the effects of prior weight [28] and type of filter. Both Gibbs- and median-type priors provided quite similar results and, therefore, in the remainder, only results with the Gibbs-type prior will be shown.

**Fig. 2:**
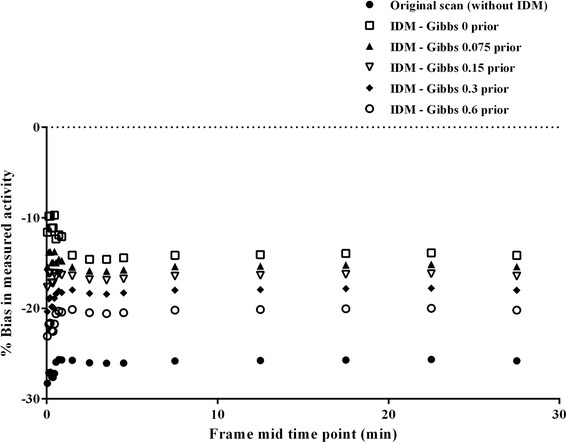
Bias in measured grey matter activity concentrations for different prior weights of the Gibbs filter

Figure 2 shows relative bias after IDM with eight iterations for varying prior weights. The figure illustrates the increase in bias with increasing prior weight. Plots also show that the lowest bias (−10% in the low-statistics frames to −14% in the high-statistics frames) was obtained when no weight (weight = 0) was applied. In addition, Additional file 2: Figure S1 shows the impact of prior weight on the noise. Standard deviation in the measurement of the Hoffman white matter activity is considered as metric for noise in the figure. Clearly, with increasing prior weight, noise decreases but quantitative accuracy deteriorates, irrespective of statistics, and hence, the prior weight should be as small as possible. Based on the data shown in Fig. 2, a prior weight of 0.15 seems to be optimal. A 0.15 prior weight resulted in only a very small negative quantitative bias with respect to a prior weight of 0 and still decreased the IDM induced noise amplification.

### Order of implementation

**Fig. 3:**
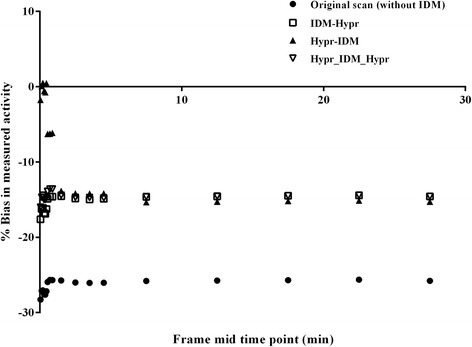
Effect of applying HYPR before or after IDM on bias in measured grey matter activity concentrations

IDM with spatial noise regularisation (i.e. IDM with prior weight > 0) partially decreased noise for high-statistics frames and had almost no improvement in SNR in the case of low-statistics frames (Additional file 3: Figure S2). Spatial noise regularisation resulted in a decrease in post-IDM quantitative accuracy and image sharpness due to its smoothing effect. Hence, implementation of HYPR was studied. The primary aim was to study the effects of the implementation order of HYPR (i.e. IDM-Hypr, Hypr-IDM, Hypr-IDM-Hypr). Figure 3 and Additional file 4: Figure S3 show results of IDM with eight iterations in combination with HYPR with different implementation orders. This figure illustrates that the order of HYPR implementation affected IDM performance, particularly for frames with low statistics. A constant activity across frames is expected, which seems to be best obtained when using Hypr_IDM_Hypr along with a decrease in bias compared with the original scan (i.e. without IDM).

**Fig. 4:**
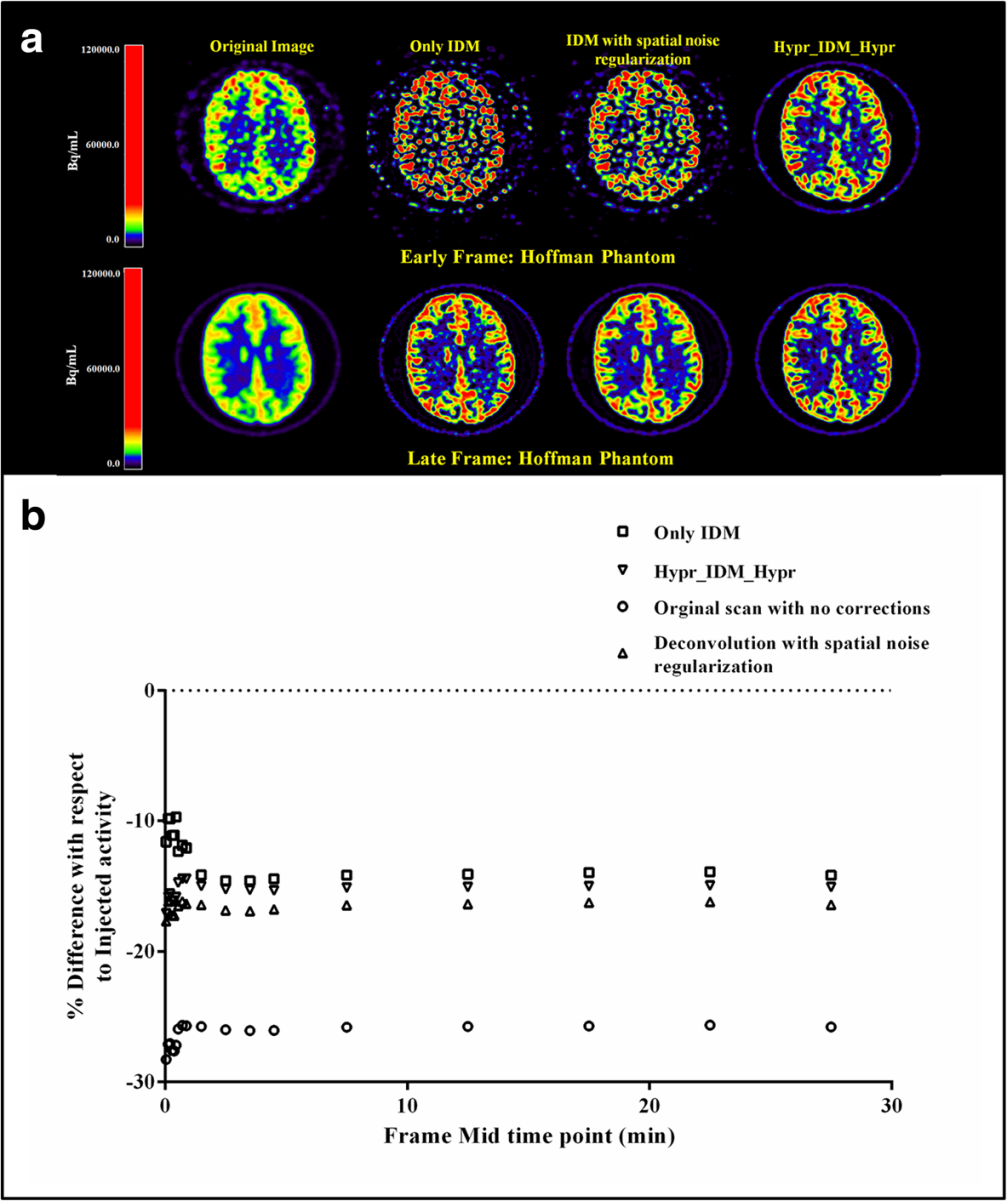
**a** Hoffman phantom images before and after applying IDM with and without spatial smoothing or HYPR for both shorter and longer frame durations. **b** Associated bias in measured grey matter activity concentrations

Figure 4 shows an example of the effects of IDM without and with HYPR or prior weight (=0.15) for frames with both low (5 s) and high (300 s) count statistics. It can be seen (Fig. 4a) that Hypr-IDM-Hypr improves image quality irrespective of frame counts. In addition, Fig. 4b clearly shows that Hypr-IDM-Hypr decreases bias from −25 to −15% as compared with the original non-PVC images.

### Clinical studies

**Fig. 5:**
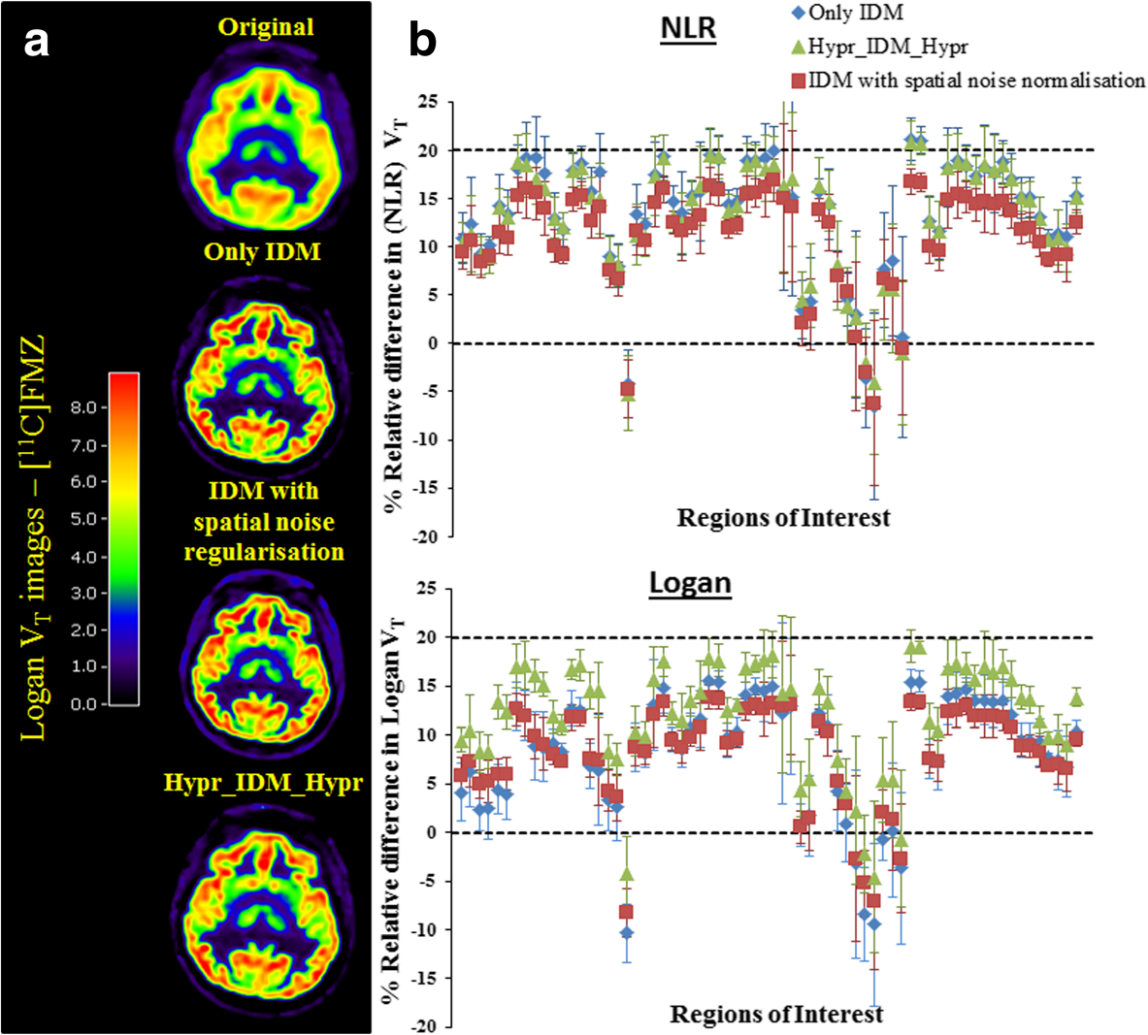
**a** Typical [^11^C]FMZ *V*_T_ images obtained using Logan analyses. **b** Quantitative effect of applying IDM on *V*_T_ values obtained using Logan and NLR

Following the phantom results, IDM with eight iterations, a spatial prior with a weight of 0.15, and HYPR pre- and post-IDM was used. Figure 5a shows *V*_T_ images of [^11^C]FMZ before and after IDM without and with HYPR or prior weight. Due to the high grey to white matter contrast for this tracer and the high count statistics, a clear increase in grey matter *V*_T_ following IDM can be observed. This is shown quantitatively in Fig. 5b for NLR and Logan analyses, respectively, with an increase between 5 and 20% depending on the VOI. Differences between the various IDM implementations, i.e. with or without prior weight or HYPR, were small.

**Fig. 6:**
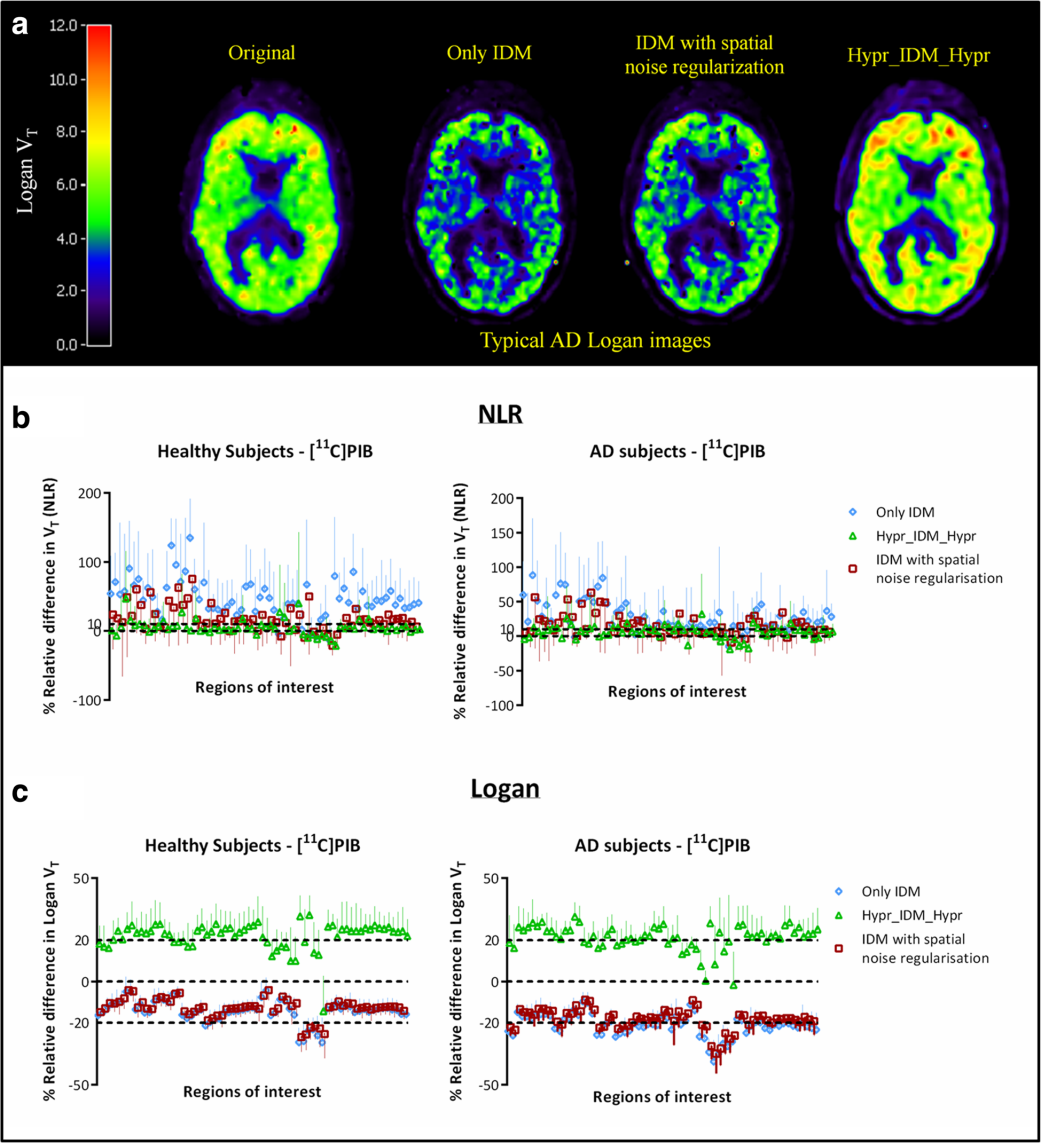
**a** Typical [^11^C]PIB *V*_T_ images for an AD subject. Quantitative effects of applying IDM on *V*_T_ values obtained using **b** NLR and **c** Logan analyses for both healthy subjects and AD patients

Figure 6 shows corresponding results for [^11^C]PIB with Fig. 6a showing Logan images for AD subjects before and after IDM. Figure 6b, c illustrate the effects of IDM on *V*_T_ obtained using NLR and Logan, respectively. In contrast to [^11^C]FMZ, both relative increases and decreases (−10 to 10%) in the NLR-derived regional *V*_T_s were observed. Interestingly, *V*_T_s obtained using Logan analysis showed a decrease with or without using the spatial priors, which was not the case for HYPR. In addition, considerable differences were observed using different implementations of IDM, which was the case for both healthy subjects and AD patients.

**Fig. 7:**
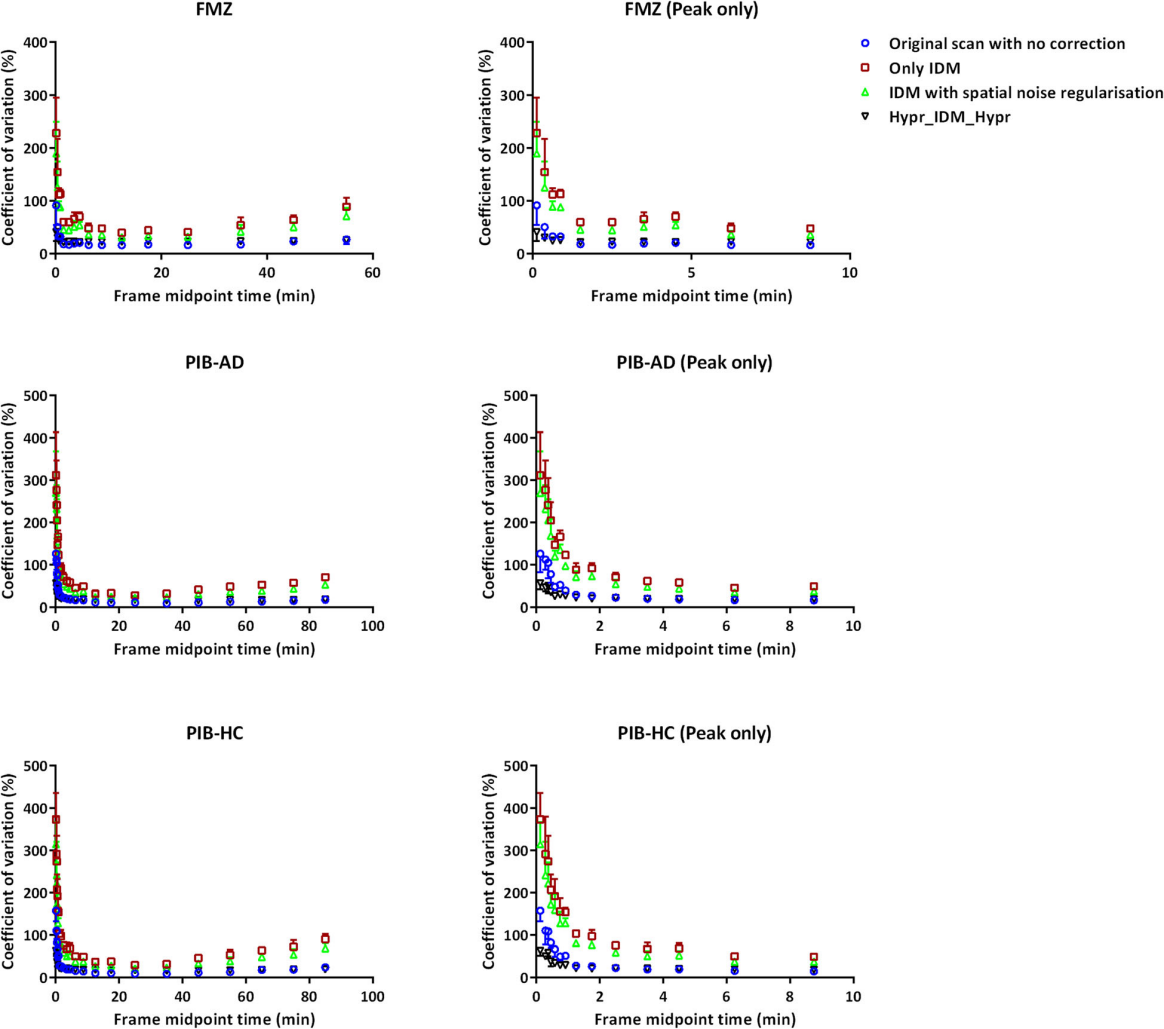
Mean ± SD of coefficient of variation (%) of voxel values within the white matter volume of interest as function of frame midpoint time for FMZ scans and AD and HC PIB scans

Finally, Fig. 7 shows the impact of different IDM implementations on image noise as function of frame midpoint time. The coefficient of variation (%) of the voxel values within the white matter VOI (size of VOI ~2.5 cc) was used as metric for image noise. The figure illustrates that only the Hypr-IDM-Hypr implementation was able to control noise to levels comparable with the original non-PVC images and that all other implementations resulted in increased image noise.

Additional file 5: Figure S4 presents the impact of the various processing schemes on a temporal lobe TAC obtained from a PIB scan of an AD patient. It illustrates the post-IDM noise amplification and denoising due to HYPR implementation. Additional file 6: Figure S5 shows that the bias (~0.087%) due to the use of HYPR is rather small at a regional level. The figure illustrates the impact of HYPR on different regions in a FMZ scan where varying regional uptake is observed. The range in the *Y*-axis of bland Altman plot represents the regional differences and the *X*-axis shows the difference in parameter estimation. FMZ scan was selected instead of PIB, to illustrate the impact of HYPR on kinetic parameter estimation without the interference of noise.

## Discussion

The phantom experiments of this study suggest that a 10–15% improvement in signal recovery can be achieved by using IDM. In addition, HYPR denoising mitigates noise amplification by IDM, without sacrificing the IDM-associated improvement in resolution. In particular, the combination Hypr-IDM-Hypr was able to improve resolution with minimal loss of precision.

In recent years, several studies [4, 10, 29] have evaluated IDM. Most of these studies have shown a reduction in PVE, resulting in quantitatively more accurate estimations of tissue radioactivity concentrations. The main disadvantage of IDM is an increase in noise, particularly for images with low statistics [19]. Regularisation of this noise can be achieved using spatial priors but at the cost of reduced PVC. Therefore, similar to Reilhac et al. [15], a denoising strategy was evaluated that can control noise with a minimal effect on spatial resolution. While in this study HYPR was used, Reilhac et al. proposed a wavelet and spectral analysis based technique. One of the possible limitations associated with the use of HYPR (with settings illustrated in this study) when compared to the implementation by Reilhac et al. is that HYPR with a single composite image (weighting factors equal for all frames) assumes that the spatial distribution of activity does not change across different time frames. If this assumption is not met, it might result in bias in TACs and subsequently in the kinetic parameter estimation. To understand the impact of HYPR, it is important to evaluate its effect on regional TACs (see Additional file 5: Figure S4), as was stated by Tohka et al. [13]. For the two tracers evaluated in this study, the use of HYPR with a single composite image resulted in a very small error on the estimation of the regional parametric values (see Additional file 6: Figure S5). However, it should be noted the use of HYPR should be validated on a study or tracer basis. The latter might not be needed using the approach of Reilhac et al. based on its fundamental properties, but we were unable to validate this. In case unacceptable biases due to HYPR would occur (e.g. for other tracer studies), its impact could be mitigated by using a moving frame composite image but possibly at the cost of less noise mitigation. In this case, the composite image will be adapted according to the changes in the activity distribution. Another advantage of the Reilhac implementation is that it can also be used for static scans, which is not the case with HYPR. A possible advantage of HYPR over wavelet and spectral analysis is that it is mathematically easier to implement and similar mathematical operations as for IDM can be used (basically only image smoothing, deconvolution and multiplication operations are needed). Both the HYPR and spectral analysis denoising strategies showed that the use of a temporal noise regularisation allowed the use of IDM without the negative effects of noise amplification on image quality. Both our and the study by Reilhac et al. showed that temporal noise regularisation methods that preserve resolution are attractive strategies for IDM implementation and result in partial volume corrections with similar noise levels as the original non-PVC data.

As explained earlier, using Hypr-IDM-Hypr, it is possible to correct for PVE while preserving SNR even in the case of low statistics (Fig. 4). This was not possible when IDM was combined with spatial noise regularisation. Although Hypr-IDM-Hypr was able to substantially correct for PVE, complete recovery was not possible (Fig. 4b). PVE also depends on the tissue fraction within a voxel (as a result of the voxel size), which cannot be corrected using IDM and HYPR. This likely explains the observed residual bias of about 10–15% in the measured grey matter activity concentrations of the Hoffman phantom.

In clinical studies, true activity concentrations and distributions within the brain are not known. Therefore, it is not possible to accurately quantify the performance of Hypr-IDM-Hypr for these scans. Nevertheless, important performance characteristics such as impact of Hypr-IDM-Hypr on regional activity, image noise and regional *V*_T_ can be derived.

[^11^C]FMZ binds specifically to central benzodiazepine receptors, which are primarily located in the grey matter regions, so in this case, one would expect mainly spill-out from the grey matter regions. Therefore, it is expected that PVC would result in an increase in grey matter *V*_T_ although the amplitude may depend on the anatomical location (thickness of the cortical rim) and the distribution of the tracer. Figure 5 shows an increase of 5–20% in regional *V*_T_ values after applying IDM. In addition, Logan images with/without HYPR or prior weight had similar visual image quality, indicating that IDM has very little impact on image quality of high statistics scans (see Logan *V*_T_ images in Fig. 5a).

[^11^C]PIB specifically binds to beta-amyloid plaques, and in this case, it is hard to anticipate the impact of PVC, as plaques are not limited to any particular VOI, so that both spill-in and spill-out may occur. Figure 6 illustrates the effect of IDM with/without prior weight or HYPR on these data sets. A clear decrease in measured *V*_T_ after application of IDM with or without spatial priors can be seen (Fig. 6c), which is not observed in the case of Hypr-IDM-Hypr. This might be due to the low SNR of [^11^C]PIB scans, where IDM without any denoising strategy may lead to further noise amplification. The change in the macroparameter of interest observed after IDM implementation also depends on the subject status. Relatively larger differences were observed for AD patients than for HC subjects. This can be expected, as tracer uptake in the grey matter is higher in AD patients, resulting in a larger partial volume effect (spill-out). Similar effects occur for spatial noise regularisation as observed in Fig. 6, particularly in Fig. 6c. Logan plots are negatively affected by low SNR [30], explaining the observed underestimation or negative bias. Due to noise, slopes of the Logan plots were negatively biased and hence resulted in an underestimation of *V*_T_. Hypr-IDM-Hypr not only corrected this underestimation but also improved SNR of the Logan images (Fig. 6a and c). In this study, the results obtained from the Logan implementation on (mainly) PIB studies were affected by two opposing factors. The first factor is the presence of noise, which results in negative bias of Logan estimated *V*_T_, particularly at high noise levels. The second effect is partial volume correction. Theoretically, PVC based on iterative deconvolution should increase *V*_T_, but it increases noise as well. Due to the fact that the noise-induced negative bias in *V*_T_ is larger than the upward correction due to PVC, an overall negative effect was observed. This was true for IDM both with and without spatial noise regularisation (i.e. use of the spatial prior). However, with HYPR, the noise was controlled and, therefore, the correction by IDM became evident. Therefore, it is essential to apply denoising before using linear methods such as Logan plots. In contrast, *V*_T_ values obtained using NLR were not affected to the same extent, possibly because NLR is performed at a VOI level, resulting in time activity curves with lower noise levels than those of individual voxels. Nevertheless, quite a few abnormally high *V*_T_ values were observed when using IDM without HYPR pre-denoising.

It is known that IDM amplifies noise in PET images, as clearly illustrated in the clinical data shown in Fig. 7. This figure also shows that none of the spatial priors were able to decrease noise substantially. However, the use of Hypr_IDM_Hypr kept image noise levels similar to those seen in the original images, i.e. without PVC.

It should be noted that although clinical scans were included in this study, the main purpose was to assess the performance of IDM in combination with HYPR. This study suggests an improvement in quantitative accuracy of PET studies after PVC along with preserved image quality using a combination of Hypr-IDM-Hypr both for low-and high-statistics scans. Hypr-IDM-Hypr may therefore be a good alternative for performing partial volume corrections of dynamic PET scans if no structural MRI scan is available.

A 6.75 FWHM for HYPR was used post-IDM to improve the SNR, and a minimal impact on the quantification was observed (Additional file 7: Figure S6) compared to using a smaller filter size that matched to post-IDM expected spatial resolution. It should be noted that the size (FWHM) of the HYPR filter should be validated for both pre and post-IDM implementation. However, we observed that using a larger filter size (larger than the expected resolution after IDM) does not notably affect the quantitative accuracy of the results, while this larger filter allowed for a better noise mitigation.

A possible limitation of IDM-based methods is that full recovery of the signal is not obtained, and a residual PVE of about 10–15% was observed. Various studies [31, 32] showed that under optimal conditions with MR image-based methods, accurate PVC can be obtained with residual errors of less than 5%. However, when MR data are used for PVC, it is of utmost importance that MR images are of high quality and that PVC methods have an accurate and reproducible performance. This was recently discussed by Greve et al. [8] who showed that the use of different PVC methods resulted in variable study conclusions. These authors recommended that one should be extremely careful in comparing results from different studies when using different PVC methods. Moreover, they discussed that MR based PVC require accurate and complete segmentation (implying that the regions with high uptake are delineated), accurate PET-MR co-registration, and homogeneous distributions within VOIs. Hence, despite the lack of full signal recovery, PET-only-based PVC approaches [15] may therefore be an attractive alternative to MR-based methods when a consistent and high quality of MR data cannot be assured and, of course, in cases when MR data are not available. In our paper, we explored the feasibility and performance of a purely PET-based PVC method in combination with HYPR denoising. Our method does not suffer from the abovementioned limitations and might therefore be a good alternative, for instance, in case of longitudinal and/or multicentre studies when MR data were collected using different systems. However, another possible limitation for this study when compared to Tohka et al. [13] is the use of Van Cittert instead of reblurred Van Cittert deconvolution. We acknowledge that the use of reblurred Van Cittert could be an attractive strategy to further improve the performance of our proposed method. Despite this limitation, we have shown that temporal noise regularisation is an attractive method for noise mitigation of iterative deconvolution-based partial volume correction, as was also shown by Reilhac et al. using a spectral analysis approach.

## Conclusions

The use of HYPR allows the application of IDM-based partial volume correction for improving the quantitative accuracy of dynamic PET studies without notably affecting SNR. Evaluations performed with clinical data confirm that Hypr-IDM-Hypr reduces PVE without notably affecting image quality.

## Additional files

Additional file 1: Algorithm for median prior. (DOCX 133 kb)

Additional file 2: Figure S1. Impact of the prior weight on the noise (standard deviation in the Hoffman phantom white matter activity measurement). (TIF 416 kb)

Additional file 3: Figure S2. Impact of the size of the prior weight on both low- and high-statistics frames. (TIF 128 kb)

Additional file 4: Figure S3. Effect of HYPR implementation before or after IDM on the low-statistics frames of Hoffman phantom images. (TIF 735 kb)

Additional file 5: Figure S4. Impact of HYPR on the temporal lobe TAC of an AD patient PIB scan. (TIF 35 kb)

Additional file 6: Figure S5. % Difference in the estimated regional *V*_T_ due to the use of HYPR on a FMZ scan. (TIF 177 kb)

Additional file 7: Figure S6. HDH implies Hypr-IDM-Hypr. A FWHM of 6.75 is used for the first HYPR and IDM, and a varying FWHM (2 or 3 mm) is used for the second HYPR. Impact of the HYPR filter FWHM used post-IDM implementation on A) Hoffman phantom, B) a FMZ clinical scan, C) an AD patient PIB scan and D) a healthy control PIB scan is presented. A) % Bias in measured activity with varying HYPR filter FWHM. Variability in the extracted regional *V*_T_ values with HYPR filter FWHM when compared to *V*_T_ values obtained from HDH 6.75-mm FWHM dataset is presented for a representative clinical scans of tracers B) FMZ, C) and D) PIB. (TIF 177 kb)
