# Supplementary material for: Partial volume correction of brain PET studies using iterative deconvolution in combination with HYPR denoising
Source: EJNMMI Res. 2017 Apr 21;7:36. doi: 10.1186/s13550-017-0284-1 (PMC5400775; doi:10.1186/s13550-017-0284-1)
Supplement: Supplementary file 1 — Algorithm for median prior. (DOCX 133 kb) [file 13550_2017_284_MOESM1_ESM.docx]

**Algorithm for median prior**: A map prior during each iteration is estimated using the following equation.

map = 2.0 * ( Image - median(Image))

The median had a neighbourhood of 1, implies that median is calculated over directly neighbouring voxels.

During the iterative update i.e while estimating the sharpened image, error image is corrected using the estimated map.

Sharpened image = Image+(error image-bheta*map)/(1.0+2.0*bheta);

bheta is used to assign different contributing weights that would determine the about of spatial filtering. Use of this prior will thus try to avoid sharp transitions of values between neighbouring voxels.
