# Supplementary figures and images for: Partial volume correction of brain PET studies using iterative deconvolution in combination with HYPR denoising
Source: EJNMMI Res. 2017 Apr 21;7:36. doi: 10.1186/s13550-017-0284-1 (PMC5400775; doi:10.1186/s13550-017-0284-1)

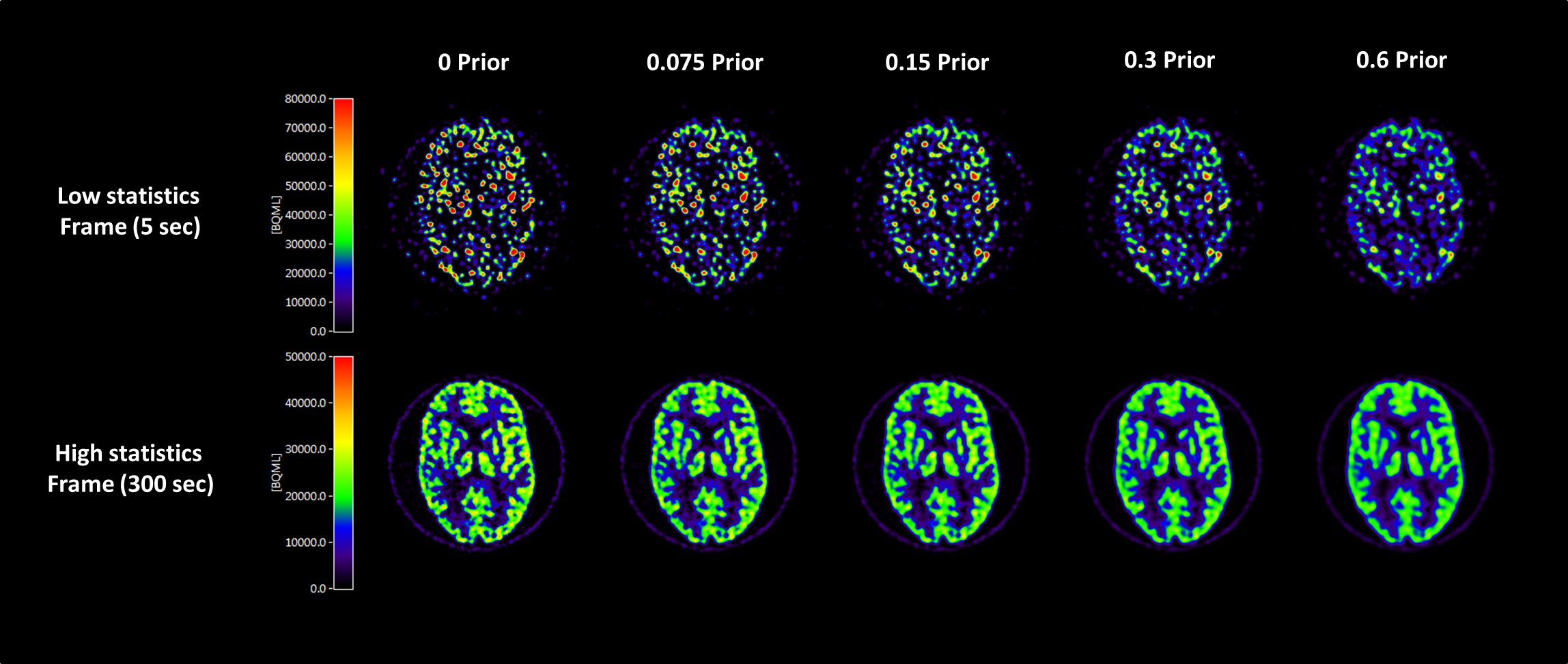

Supplement: Supplementary file 3 — Impact of the size of the prior weight on both low- and high-statistics frames. (TIF 128 kb) [file 13550_2017_284_MOESM3_ESM.tif]

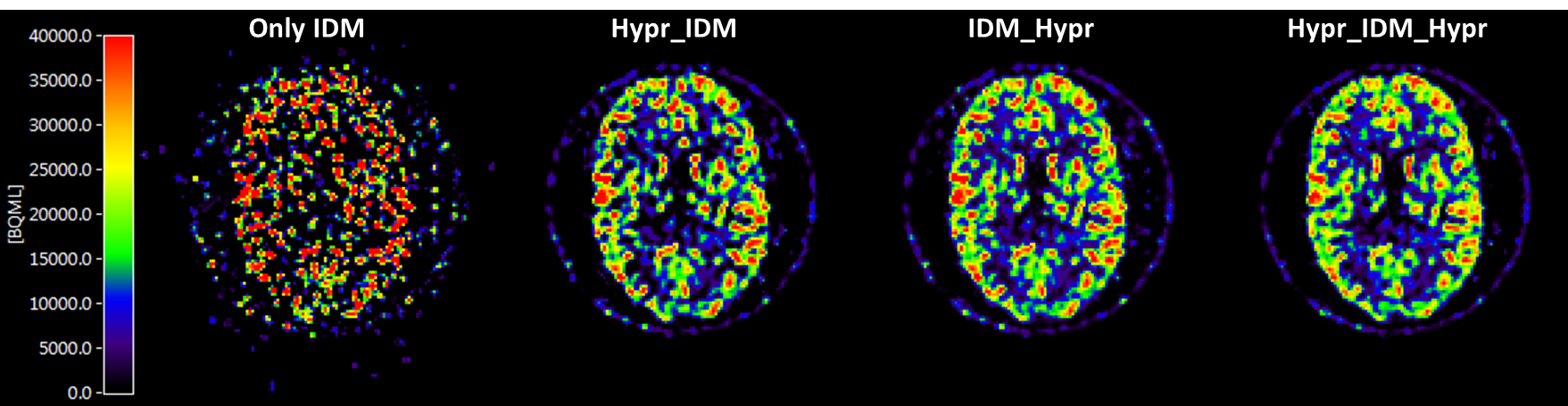

Supplement: Supplementary file 4 — Effect of HYPR implementation before or after IDM on the low-statistics frames of Hoffman phantom images. (TIF 735 kb) [file 13550_2017_284_MOESM4_ESM.tif]
